# Oncofertility and Reproductive Counseling in Patients with Breast Cancer: A Retrospective Study

**DOI:** 10.3390/jcm11051311

**Published:** 2022-02-27

**Authors:** Simona Zaami, Rossella Melcarne, Renato Patrone, Giuseppe Gullo, Francesca Negro, Gabriele Napoletano, Marco Monti, Valerio Aceti, Alessandra Panarese, Maria Carola Borcea, Chiara Scorziello, Luca Ventrone, Samira Nicole Mamedov, Maria Letizia Meggiorini, Massimo Vergine, Laura Giacomelli

**Affiliations:** 1 Department of Anatomical, Histological, Forensic and Orthopedic Sciences, “Sapienza” University of Rome, 00161 Rome, Italy; francesca.negro@uniroma1.it (F.N.); gabriele.napoletano@uniroma1.it (G.N.); 2 Department of Surgical Sciences, “Sapienza” University of Rome, 00161 Rome, Italy; rossella.melcarne@uniroma1.it (R.M.); mariacarola.borcea@uniroma1.it (M.C.B.); chiara.scorziello@uniroma1.it (C.S.); ventrone.1639886@studenti.uniroma1.it (L.V.); mamedov.1825421@studenti.uniroma1.it (S.N.M.); massimo.vergine@uniroma1.it (M.V.); laura.giacomelli@uniroma1.it (L.G.); 3 ICTUS, University of Naples Federico II, 80131 Naples, Italy; dott.patrone@gmail.com; 4 In Vitro Fertilization Unit, Department of Obstetrics and Gynecology, Villa Sofia Cervello Hospital, University of Palermo, 90146 Palermo, Italy; gullogiuseppe@libero.it; 5 Department of Maternal and Child Health and Urological Sciences, Policlinico Umberto I, “Sapienza” University of Rome, 00161 Rome, Italy; marco.monti@uniroma1.it (M.M.); marialetizia.meggiorini@uniroma1.it (M.L.M.); 6 Department of Translational and Precision Medicine, “Sapienza” University of Rome, 00161 Rome, Italy; valerioaceti@gmail.com; 7 General and Transplant Surgery Department, Dipartimento di Scienze Cliniche Applicate e Biotecnologiche (DISCAB), University of L’Aquila, 67100 L’Aquila, Italy; alessandra.panarese@univaq.it

**Keywords:** breast cancer, oncofertility, counseling, risk factors, medicolegal issues

## Abstract

Introduction. Improving the prognosis of breast cancer patients is of utmost importance in terms of increasing survival rates. Modern medicine has therefore prioritized better quality of life for patients, even after the disease, through a better management of the potential long-term side effects induced by anticancer treatments. Fertility preservation and family planning are therefore crucial issues to be addressed in all cancer patients of reproductive age. Along those lines, a new branch of medicine with distinct multidisciplinary characteristics has developed over the years: oncofertility. Although both national and international guidelines value reproductive counseling as an essential aspect of the diagnostic-therapeutic pathway, part and parcel of the informed consent process, it is not included within the protocols adopted by the operating units for the care and management of neoplastic diseases. Objective. This study aimed to evaluate the activity of the Breast Unit of the Policlinico Umberto I Hospital, Rome, Italy, and the degree of compliance with guidelines. By knowing the strengths and weaknesses of such approaches, the standards of care offered to breast cancer patients can be improved. Materials and methods. A retrospective study based on a review of medical records was conducted between 2014 and 2021. Patients under 40 years of age diagnosed with non-metastatic malignancies were included who received chemotherapy treatment, namely neoadjuvant, adjuvant or adjuvant hormone therapy. Results. The data were extracted from the medical records of 51 patients who met the inclusion criteria, 41% of whom received reproductive counseling, and of these, 43% decided to undertake a path of fertility preservation. Factors such as the absence of children and young age reportedly favored both the interest in counseling proposals by the medical staff and the decision to undertake a path of fertility preservation. Conclusions. The study shows that there has been growing interest in the topic of oncofertility, especially in light of law 219/2017. Therefore, since 2018, multiple proposals for reproductive counseling have been set forth, but there was not an equally growing demand for fertility preservation practices, which can be explained by the invasive nature of such practices, the patients’ concern about their own state of health, and poor or inadequate information. Such impediments highlight the importance of standardized counseling and the need for a multidisciplinary medical team to support the patient in the decision-making process. The study also revealed a drop in the number of patients receiving counseling due to the COVID-19 pandemic, contrary to the positive trend that was recorded prior to the pandemic.

## 1. Introduction

Breast cancer is the most common malignancy occurring in young women, who account for 7% of the total number of breast cancer cases diagnosed each year in Western countries [1,2]. Most available data point to young age having negative prognostic relevance at the time of diagnosis. Therefore, young patients are often candidates to be managed with aggressive multimodal therapies that seriously jeopardize fertility. Breast cancer, according to the data presented in the 2019 AIOM-AIRTUM report (Italian Association of Cancer Registries) [3], represents the most frequent neoplasm in women of any age, with 30% of cases of the total diagnosis. Furthermore, in the 0–49 age group, its incidence reaches 40% even though such an age group has the lowest incidence. The AIOM guidelines for breast cancer [4], using the 2018 estimates, highlight a slightly increasing trend in the annual incidence (+0.3%), coupled with an average drop in mortality on a yearly basis (−0.8%) which, in the younger groups, rises to 0.9%. The shift in the minimum age to qualify for mammograms in some regions contributes significantly to the increase in incidence in the 45–49 age group, with a consequent increase in early diagnoses, which explains the parallel decline in mortality mentioned above.

Breast cancer is the leading cause of cancer-related deaths in women, with a survival rate standing at 87% in Italy overall, which has remained steady in the 15–75 age group, while it is lower in patients over 75. The age bracket accounted for in this study (15–44 years) has a 5-year survival rate of 91%. Although there is a North-South discrepancy in incidence, with the North being at a higher risk (probably due to environmental factors), the survival rate is higher in the North than in the South, possibly due to the less efficient regional healthcare systems in Southern regions [5].

In absolute terms, there are about 800,000 women with a previous diagnosis of breast cancer. From what has emerged so far, it seems safe to assume that taking care of the underlying pathology is not enough in patients with breast cancer; in fact, it is recommendable, both from good clinical practice and from an ethical point of view, to devote the right attention to the quality of the aftercare life.

### 1.1. Histological and Molecular Classification

The diagnosis of breast cancer relies on two different tests aimed at evaluating the nature of the lesion. In 2012, with a recent 2021 update, the World Health Organization (WHO) laid out the histological classification [6,7] of breast cancer. Numerous different histotypes are known in the literature; however, in clinical practice, the most common is invasive ductal carcinoma, with a 70% to 80% frequency. Another purpose of the biopsy examination is the staging of the lesion based on the TNM system, which is based on three parameters:

- Primary tumor (T), wherein the local extension of the tumor is examined;
- Regional lymph nodes (N), wherein the impairment of the draining lymph nodes are assessed. In the case of the breast, the axillary lymph nodes are of primary interest;
- Distant metastasis (M), werein the presence or absence of systemic metastases is studied.

The purpose of staging according to the TNM system is to facilitate clinical decisions and an accurate clinical evaluation, so as to be able to set the best therapeutic path, combining maximum benefits with less stress for the patient. Gauging the local extension of the tumor is fundamental for planning the surgical removal, which must guarantee total eradication of the lesion by exporting as little healthy tissue as possible. Local extension or the involvement of the regional lymph nodes is a useful parameter for an oncological evaluation as to when to start radiotherapy or chemotherapy to eliminate any neoplastic foci. Over time, clinicians have come to realize that although the clinical manifestations and stage of disease were equivalent, patients did not respond in the same way to therapy. In light of such clinical evidence, research has then focused on the molecular profile of breast cancer. Biological investigations have led to the the classification of invasive carcinoma into four subtypes [8,9,10,11,12]:

- Luminal A, neoplasms with the expression of hormone receptors for estrogen and progesterone, associated with HER2 negativity and low Ki67 levels [13];
- Luminal B, neoplasms that express hormone receptors, like Luminal A, which are associated with high values of proliferative activity. These, in turn, are divided according to the expression of HER2 into HER2 negative and HER2 positive, in which the levels of Ki67 are not relevant, the strong replicative activity is at the basis of a high risk of recidivism [14];
- HER2-positive, highly expressed HER2 (3+ in immunohistochemical reactions) with an absence of estrogen receptors [15];
- Basal-like or triple-negative, absence of both receptor expression for estrogen and progesterone and HER2, matched by high basal cytokeratin levels [16,17].

Thanks to this classification, the behavior of individual breast cancer subtypes can be assessed in relation to the risk of metastasis [18,19]. The presence of estrogen receptors was found to be a favorable prognostic factor, and the triple-negative subtype is the more aggressive variant. From a clinical standpoint, each subtype has a different metastatic dissemination pattern.

#### Treatment of Breast Cancer

Since they are based on each patient’s therapeutic path, countermeasures must be taken to protect the quality of life in the long term. The initial development of the surgical practice was driven by the will of the medical community to counter the major cause of distress and post-operative psychological outcomes: the demolition of the patient’s breast with evident aesthetic damage that can compromise the woman’s sense of femininity. Currently, radical mastectomy is only carried out in advanced cases with adverse prognosis [20,21]. Although surgery is not the main focus of this article, which is centered around the type of non-surgical therapies adopted in the various stages of cancer, the surgical element is still highly relevant in terms of defining the difference between neoadjuvant and adjuvant therapies (the former take place before surgery, while the adjuvant therapies follow it). Ductal carcinoma in situ (DCIS) is a pre-invasive lesion with the potential to evolve into invasive carcinoma and represents the first stage of malignant breast cancer [22,23,24]. Radiotherapy of the operating field is usually considered. In case of positivity to the immunohistochemical tests for estrogen receptors, tamoxifen can be administered. Surgery alone does not significantly reduce the risk of recurrency [25,26]. As previously observed, hormone therapy causes a risk of infertility [27,28] which, although lower than that of most chemotherapy drugs, must be taken into account in the oncological consultation, particularly for patients of childbearing age. In fact, the risk for infertility lead to a decrease in the use of tamoxifen in this group of age. There is no evidence to support chemotherapy in the treatment of DCIS.

Lobular carcinoma in situ (LCIS) is considered a benign lesion, although it can be associated with an increased incidence of both ipsi- and contra-lateral invasive breast cancer [29]; therefore, it does not require any neoadjuvant or adjuvant treatment.

The treatment of infiltrating carcinoma [29], if immediately operable, requires subsequent conservative surgery, adjuvant radiotherapy, the administration of adjuvant chemotherapy and hormone therapy. Moreover, in ER- and progesterone receptor (PgR)-positive histotypes, biological drugs combined with each other must also be evaluated [30].

Neoadjuvant chemotherapy is used, particularly for most cancers larger than 2 cm, possibly associated with biological drugs. The purpose of this therapeutic approach is to reduce the size of the carcinomatous lesion, favoring less impactful forms of surgery and ensuring better aesthetic results and fewer post-operative complications. Response to adjuvant chemotherapy is a favorable prognostic index. For carcinoma that is not primarily operable, neoadjuvant chemotherapy treatment is instrumental in allowing subsequent surgery. The response to neoadjuvant chemotherapy in this case becomes a favorable prognostic factor.

Only 5% of tumors are diagnosed in the metastatic phase, and when it happens, patients routinely undergo a thorough multidisciplinary evaluation of their general health conditions. Only in 2–3% of such patients can long-term survival or even recovery be the main objective [31,32].

These patients undergo chemotherapy cycles, often as polytherapy; thus, they are at high risk of infertility. In addition, patients who have to undergo neoadjuvant chemotherapy should be able to rely on timely reproductive counseling in order to avoid delays in the initiation of therapy that could result in worse prognosis.

### 1.2. Antineoplastic Treatments and Infertility

Infertility resulting from antineoplastic treatments can be determined by radiotherapy, cancer surgery and chemotherapy. There are two mechanisms by which anticancer therapies may interfere with a woman’s reproductive capacity:

1. Direct damage due to destruction of primary follicles that cannot be replaced, resulting in premature ovarian exhaustion (POF) as the ovarian reserve has been significantly reduced by the treatment;
2. Indirect damage that can result either from the involvement of granulosa cells with hormone production deficiency and, therefore, the development of temporary hypoestrogenic hypergonadotropic amenorrhea lasting about 2 months with the recovery determined by the entry of new follicles in the cyclic phase or due to the compromise of the vascular network and of the ovarian stroma with ischemic suffering of the primordial follicles, which causes the apoptosis of the latter [33,34].

Antineoplastic drugs can be subdivided into five classes, according to the frequency of the aforementioned adverse events:

- High risk (>80%): cyclophosphamide, adjuvant therapy for breast cancer in combination with methotrexate, fluorouracil, doxorubicin, epirubicin in patients >40 years;
- Intermediate risk (20–80%): taxani, adjuvant therapy for breast cancer in combination with methotrexate, fluorouracil, doxorubicin, epirubicin in patients 30–39 years;
- Low risk (<20%) vinblastine, bleomycin, dactinomycin, 6-mercapto-purine, adjuvant therapy for breast cancer in combination with methotrexate, fluorouracil, doxorubicin, epirubicin in patients >30 years;
- Very low/absent risk: vincristine, 5-fluorouracil, methotrexate;
- Unknown risk: oxaliplatin, irinotecan, monoclonal antibodies, tyrosine kinase inhibitors [34,35,36,37].

Such a classification notwithstanding, the risk of iatrogenic infertility is difficult to assess at the beginning of treatment: the presence of menstruation is not a reliable parameter, albeit used as an index of fertility by numerous studies, as it may underestimate the ovarian reserve and the woman’s capacity to bring a pregnancy to term. A Chinese study on the risk of chemotherapy infertility, in fact, highlights how the percentage of post-chemotherapy women with low levels of anti-Müllerian hormone (AMH) [38,39] is higher than those who have experienced transient amenorrhea [40]. Regardless of the therapeutic regimen adopted, the greatest risk factor for iatrogenic infertility is the patient’s age, with a directly proportional correlation. In women under the age of 35, the risk of POF (premature ovarian failure) is about 10%. It rises to 50% between the ages of 35 and 40, and reaches 85% over the age of 40 [41,42]. Although for younger patients there is a lower risk of undergoing POF, given the greater ovarian reserve, the possibility of future damage must be taken into account. It is therefore of great importance to protect all public administrations clients, as the relationship between risks and benefits is totally in favor of the latter.

A further threat to the patient’s fertility is the use of adjuvant hormone therapy to prevent relapses in cases of hormone-responsive cancers, such as most breast cancer. Tamoxifen, for example, a selective estrogen modulator, has a receptor antagonist activity that blocks the growth of some types of neoplastic diseases, although by doing so it interferes with the endocrine function of the ovary, causing a deficit of reproductive function and increasing the risk of iatrogenic infertility from chemotherapy, if given in combination [43,44,45]. Highly relevant studies such as the Pregnancy Outcome and Safety of Interrupting Therapy for Women With Endocrine-Responsive Breast Cancer (POSITIVE) have been undertaken to investigate whether temporary discontinuation of endocrine therapy for the purpose of allowing pregnancy may entail a higher risk of breast cancer recurrence [46]. Certainly, patients who wish to achieve motherhood following breast cancer treatment ought to consider that 5–10 years of endocrine therapy (ET) could negatively affect the likelihood to have a pregnancy. Nonetheless, no prospective study has so far involved a shorter ET run in this population. Ultimately, although birth outcomes following breast cancer have been reported to be no different than those among the general population, higher risks have been found regarding delivery complications, preterm birth, low birth weight and Cesarean section [47].

A recently introduced therapeutic alternative is based on monoclonal antibodies, molecules directed towards specific tumor antigens. Although they have not been studied enough for possible side effects on female fertility, an early multicenter clinical trial seems to suggest that monoclonal antibodies are not associated with iatrogenic amenorrhea [48].

### 1.3. Oncofertility

Fertility preservation in cancer patients has been gaining ever greater attention, largely thanks to the progress in cancer care with increasingly effective chemo-radiotherapy protocols leading to significantly higher survival rates and life expectancy.

Oncofertility is the branch of medicine born from the synergy of oncology and fertility preservation procedures. Its primary objective is to study, outline and implement the most advanced techniques to preserve the patients’ fertility and reproductive potential [49]. The high incidence of neoplasms in individuals of childbearing age, along with longer life expectancy rates, has prompted health care professionals to pay ever greater attention to the psycho-physical well-being and therefore to the quality of life of patients in the stages following the disease. The increasing incidence of neoplastic diseases in childbearing age is also a financial burden for National Health Systems in terms of care management and long-term follow-up. The World Health Organization (WHO), in an analysis of data from 2015, highlighted that, worldwide, there are 14 million survivors following a diagnosis of cancer, of which about 5% (700 thousand) are under 40 years of age [50]. As for Italy, the joint report of the Italian Association of Medical Oncology (AIOM) and the Italian Cancer Registries Association (AIRTUM) 2019, published on the Ministry of Health website, estimates that new cancer diagnoses amount to 371 thousand, with a decrease, therefore, of about 2000 cases compared to the previous year, in the female sex. On the other hand, there is an increase in breast tumors and, in contrast to the male sex, also in pulmonary neoplasms. This is due to an increase in smoking in women of all ages. The 5-year survival percentage has increased, reaching a rate of 63% for women and 54% for men, resulting in a consequent increase in those who live after diagnosis, who number almost 3.5 million [1]. For the purposes of this report, the main analysis was focused on the female population, where various critical issues emerged compared to the male counterpart, as reported by a British study [51], in light of the anatomical and physiological differences that complicate the process of preserving fertility in women and the different methods applied for this purpose.

Available strategies for ovarian function and/or preservation of fertility in young breast cancer patients prior to administration of chemotherapy include ovarian suppression with gonadotropin-releasing hormone (GnRHa) agonists during cytotoxic therapy, cryopreservation of oocytes and embryos and the cryopreservation of ovarian tissue.

#### Preservation of Fertility in Breast Cancer

There are many techniques available for the preservation of fertility in cancer patients. The first distinction must be made based on the patient’s sex for obvious anatomical and physiological reasons. Furthermore, not all techniques are viable for every type of neoplasm.

For example, for breast cancer, given the well-circumscribed field of irradiation, it is not necessary to take precautions against the gonadotoxic effects of radiotherapy. At the present time, there are three main strategies that are adopted to preserve fertility in this class of women. Embryo freezing is illegal in Italy under law 40/2004, mainly on ethical grounds, despite being a well-established and effective practice [52,53]. Cryopreservation of oocytes is the standard technique for the preservation of fertility in women, and since 2013, it is no longer considered an experimental technique [54,55].

The method involves three phases:

1. Induction of multiple follicular growth: this entails an ovarian stimulation phase, obtained through the daily subcutaneous injection of gonadotropins, associated with the subcutaneous injection of a similar luteinizing hormone-releasing hormone (LHRH) to avoid early spontaneous ovulation. The duration of the stimulation can vary between 9 and 15 days, causing a delay in the start of chemotherapy;
2. Ultrasound-guided egg retrieval: this consists of a short-term invasive procedure, which can be performed under general or local anesthesia. Complications are rare;
3. Evaluation, selection and cryopreservation of oocytes: this is a laboratory phase in which the collected oocytes are processed. Those in metaphase II are selected for cryopreservation through vitrification [56,57,58].

At present, given the lack of studies conducted on large samples, little is known about the effectiveness of the technique in terms of completed pregnancies. The first data that emerged are very satisfactory [59].

Cryopreservation of ovarian tissue, on the other hand, is a technique considered experimental; however, it can be useful in cases where it is not possible to postpone the start of chemotherapy. A review published in 2017 reports more than 80 cases of full-term pregnancies following ovarian tissue transplantation [60]. The management and clinical outcomes of breast cancer survivors who managed to achieve pregnancy and patients diagnosed with pregnancy-associated breast cancer (PABC) have been elaborated on in great detail in the 2017 PREgnancy and FERtility (PREFER) study, a broad-ranging prospective cohort study encompassing several Italian hospitals linked to the Gruppo Italiano Mammella (Italian Breast Group, GIM). Interestingly, the study points out how a degree of safety in terms of the pregnancy in cancer survivors has been proven by available retrospective findings. That holds true for patients suffering from hormone receptor-positive disease [61].

It is also worth remarking that according to the same study, neonatal outcomes in cancer survivors are apparently similar to those among the general population, although higher abortion and birth complication rates were reported in the former group as well [62].

### 1.4. Rules Governing Oncofertility

The fundamental goal pursued by oncofertility is in keeping with inalienable rights enshrined in international treaties and covenants.

In 1948, the General Assembly of the United Nations in art. 16 of the Universal Declaration of Human Rights stated: “men and women of full age have the right to marry and to found a family, without any limitation due to race, citizenship or religion […]” [63].

In 1950, the European Convention on Human Rights added to Article 8: “Everyone has the right to respect for their private and family life […] and there can be no interference by a public authority in the exercise of this right, unless such interference is required by law [64]”.

The 1968 International Conference on Human Rights in Tehran, on the other hand, represents the first international document that establishes the right to self-determination of the family. In paragraph 16, it states: “The protection of the family and the child remains the goal of the international community. Parents have the fundamental human right to freely and responsibly determine the number of children and the time lapse between one child and another” [65].

In 1994, with the International Conference on Population and Development, held in Cairo, a turning point was reached with an international acknowledgment of the importance of sexual and reproductive rights [66]. The Action Program that emerged stated: “Reproductive health is a state of complete physical, mental and social well-being—And not simply an absence of disease or infirmity—On all aspects relating to the reproductive system, its processes and functions. Reproductive health therefore implies that people have a satisfying and safe sex life, that they have the ability to procreate and the freedom to decide if, when and how often”. The forerunner in the reception of international declarations is the USA, where the Oncofertility Consortium was founded in 2005 to implement the union between endocrinological-reproductive needs and treatments at risk of infertility, especially in young women [67].

In Italy, with the promulgation of Law 40/2004, the State regulates access to medically assisted procreation (MAP) paths guaranteed by the National Health System; however, the restrictions imposed are deemed too strict and ineffective in upholding the rights of all citizens.

Therefore, on 18 June 2014, the Constitutional Court declared the legitimacy of using heterologous fertilization techniques; the following year, a Constitutional Court ruling legalized access to medically assisted procreation techniques for fertile couples who are carriers of transmissible genetic diseases, thus expanding the population to be granted free access to MAP. Furthermore, questions of legitimacy were also raised with regard to other articles of law 40/2004, declaring it in fact an incomplete law, ill-suited to the current needs of the Italian population.

In 2016, following a joint statement of purpose from three different associations, AIOM (Italian Association of Medical Oncology), SIE (Italian Society of Endocrinology) and SIGO (Italian Society of Gynecology and Obstetrics), a new set of recommendations was released, which stressed the need for a national network of oncofertility centers (OCs), highly specialized facilities in a limited number but rationally placed on a regional basis, in order to provide an accessible and efficient service to the entire population. The above-mentioned document also recommends the creation of an oncofertility center in each region that brings together doctors of various specializations, oncologists, gynecologists, reproductive doctors and also psychologists and biologists to establish the best approach for the patient, both from a technical and human point of view. Consultancy should be provided by the local centers to which citizens turn to for the diagnosis and treatment in order to provide the most effective and updated diagnostic-therapeutic path [68]. To achieve this goal, the recommendations lay out a set of criteria which should be met by OCs:

- They should be placed within public health facilities that meet the multidisciplinary criteria and the criteria of structural compliance with the Guidelines;
- They ought to be capable of guaranteeing a dedicated and consistent service and be adequately staffed (gynecologists, endocrinologists-andrologists, biologists-oncologists, psychologists and nurses);
- OCs need to rely on an effective booking system with availability for specialist-patient consultation within 24–48 h on an informative website;
- OCs need to be able to provide adequate counseling on cryopreservation, on any subsequent MAP options and techniques with adequate information material, and create a standardized informed consent form and digital archiving, preferably on a national basis.

The administration’s interest is reflected in the 2015 National Fertility Plan issued by the Ministry of Health and the inclusion of outpatient and specialist services among the Essential Levels of Assistance (LEA) for couples requiring medically assisted and autologous procreation that are heterologous, and, at the same time, the collection and conservation of donated gametes for the purpose of heterologous fertilization are guaranteed [69].

### 1.5. Reproductive Counseling in Cancer Patients

Reproductive counseling refers to the phase in the doctor-patient relationship in which the risks of chemotherapy treatment on the reproductive system’s health are discussed, in addition to the best medical options for preserving fertility. The purpose of well-conducted counseling is to help patients overcome both the psychosocial discomfort deriving from a diagnosis of infertility, temporary or permanent, and to envisage a better quality of life after the disease, as also recommended by the guidelines of the American Society of Clinical Oncology (ASCO) [36,37] and the already mentioned Italian Association of Medical Oncology (AIOM) [68]. Therefore, it is essential that all healthcare professionals dealing with cancer patients of childbearing age are adequately prepared to provide information on the risks of likely impaired reproductive endocrine capacity related to anticancer care and to illustrate all options for preserving fertility.

The guidelines emphasize that the discussion on these topics should be an integral part of the specialist assessment and of doctor-patient counseling as a whole, while pointing out that often, the fertility issue is neglected, which means we are still far from a systematic and timely application of counseling, according to the provisions of the Barcelona Recommendations [70]. This often deprives patients of access to fertility preservation treatments.

Fertility, for a woman, represents much more than the ability to give birth: it is a sign of femininity and womanly identity, which may at times lead to a presumption of infertility due to the inadequate information received during the diagnostic-therapeutic process [71,72]. On the other hand, although the effects that anti-tumor therapy has on reproductive capacity are well known and widely documented, not all women facing a diagnosis of cancer are aware of the long-term consequences. Therefore, counseling and providing information for the patient is important not only for the purpose of fertility preservation; it also allows her to acknowledge the course of her disease, and to become aware of its outcomes [73].

A US study conducted on a group of young cancer patients reveals that over 70% of those who, at the time of diagnosis, had no children yet, wished to become parents in the future, and, moreover, most of them believed that the experience of the disease could make them better parents [74]. From what has been reported so far about the risk of infertility from chemotherapy and the importance that this side effect plays in the psychology of patients who survived the disease, it can be evidently seen how the doctor who takes care of the health of this population segment has a great responsibility both in terms of the physical and mental health of the person concerned. It is in fact of utmost importance to articulate the communication process with the right timing and approach so that the patient is receptive, responsive and ultimately capable of making a reasoned, conscious and free choice as to the most suitable therapeutic procedures to be undertaken [75].

Reproductive counseling, therefore, needs to take on a rather wide-ranging value: it has to be coordinated with the other phases of the diagnostic-therapeutic path, so as not to burden the patient with unnecessary concerns, and inform them as to the best path to be taken both for the treatment of neoplastic diseases and the restoration of acceptable quality of life [76]. Given the complex context in which the medical team operates, and despite the undeniable progress that many health systems have made, much work still needs to be done to ensure the best health care is available to young cancer patients. Still, the early involvement of a specialist in reproductive medicine, with the theoretical and practical skills to conduct an accurate reproductive counseling phase, is currently not part of routine clinical practice [77]. It is the duty of health professionals to provide information as to the clinical status, the possible therapeutic pathways and all the viable options for preserving fertility in a thorough, understandable fashion. The entire medical team should make a concerted effort to get through to their patients, striving to meet their needs regardless of social background, level of education and religious belief. Still, even greater attention is necessary when catering to the needs of cancer patients, since such a future risk is often not even conceived by the young patient. At the same time, it is necessary to prepare the patient to undertake a therapeutic choice in a relatively short time [78]. The essential role played by reproductive counseling is now widely recognized by the medical community, but still, much remains to be done in order to achieve complete coverage of all patients of childbearing age diagnosed with cancer. A German retrospective study reports that in 20 years, the rate of patients who remembered having engaged in a discussion about the risk of iatrogenic infertility went from a third, in the period between 1980 and 1984, to half in the four years 2000–2004 [79,80]. An Australian review published in May 2019 [81] has collected more than 30 documents drawn up by 19 national and international organizations from Europe and much of the Western world. This list of documents consists of guidelines, clinical recommendations and expert opinions representing a cross-section of the various health systems which, despite the differences between their fundamental principles, agree on the relevance and value of reproductive counseling for fertility preservation. In analyzing the current international state of affairs, it is worth mentioning the 2018 guidelines by the American Society of Clinical Oncology (ASCO) [37], which decidedly reaffirm that the dialogue on risks and solutions for the preservation of fertility must be prioritized within the diagnostic-therapeutic process for both oncologists and other healthcare professionals. To better achieve the goal of providing adequate counseling, the guidelines lay the foundations for the correct management of patients of childbearing age diagnosed with cancer who risk compromising their reproductive capacity permanently, through three recommendations for doctors:

- Recommendation 1: cancer patients are to be considered interested in discussing the preservation of their fertility. It is, therefore, the duty of the doctor and in general of any health professional to talk about the risk of infertility as early as possible before treatment;
- Recommendation 2: healthcare professionals should refer patients who express an interest in preserving fertility (and those who are undecided) to a reproductive specialist;
- Recommendation 3: in order to keep all options open, fertility preservation ought to be discussed as early as possible before the start of therapies.

A thorough and exhaustive discussion can ultimately reduce suffering and improve the quality of life. A second consultation may be necessary upon the patient’s return for follow-up after completion of therapy and if the patient has considered the desire to become pregnant. The entire counseling process should be reported in the medical record. Recommendation 2 highlights the importance of a specialist in reproductive medicine, which is often underestimated. Referral for second-level counseling to a professional expert in the subject enables the patient to receive much more detailed information compared to what might be offered by the oncologist or surgeon. Furthermore, the dialogue would take place in a different setting that does not focus on the diagnosis of cancer but on the patient’s well-being. Still, reports show that this approach is not sufficient to reach the patient’s full awareness. Hence, in addition to counseling by a reproductive medicine specialist, the support of paper or digital material freely available by the patient is advisable, so that patients can absorb at their own pace and outside the health facilities the large amount of information received. Moreover, the intervention of a psychosocial consultant should be considered as well to help patients deal with the psychological distress that diagnosis and therapy certainly entail [82].

In conclusion, reproductive counseling is universally recognized as an essential phase in the diagnostic-therapeutic process of the cancer patient, resulting in undeniable benefits in terms of health and quality of life. However, several issues linger, including, first of all, the lack of information from doctors who treat patients of childbearing age. A recent Italian study highlights the lack of knowledge of oncologists on the subject [83,84], and the shortage of reproductive specialists within the multidisciplinary oncological-surgical teams. Another element that complicates the doctor-patient dialogue is precisely the gender of doctors and of patients: male doctors have been found to have more ease in relating to patients of the same sex, which causes gender disparity, since it is female patients who generally need counseling the most [85].

## 2. Experimental Study

### 2.1. Objectives of the Study

In recent years, the interest of the scientific and clinical world has grown in the prevention of reproductive capacity in young women diagnosed with cancer as a consequence of the gonadotoxicity resulting from anti-cancer treatments.

Although scientific literature largely agrees on the importance of reproductive counseling, diffusion in clinical practice is still insufficient.

The objectives of our study are to:

- Investigate adherence to good clinical practice, as dictated by the guidelines, within the Breast Unit of the Policlinico Umberto I in Rome, evaluating the number of patients with whom reproductive counseling took place;
- Analyze the relationship between the diagnosis and the fertility preservation proposal;
- Investigate the discrepancy between the counseling proposal and adherence to fertility preservation practices;
- Investigate the ways in which reproductive counseling is conducted and the patient’s satisfaction in this regard;
- Analyze the impact of the SARS COVID-19 pandemic on the issue of oncofertility;
- Raise awareness and inform doctors on the topic of “oncofertility” and on the importance of reproductive counseling in the multidisciplinary management of cancer patients of childbearing age for the fundamental purpose of promoting adherence to national guidelines for the preservation of fertility in cancer patients, in compliance with the legal and ethical duties of the doctor.

### 2.2. Materials and Methods

#### 2.2.1. Study Design

This is a retrospective study based on the analysis of medical records compiled by the Breast Unit of the Policlinico Umberto I in Rome for the 8-year period from 2014 to 2021.

#### 2.2.2. Procedure and Methods of Data Collection

The selection of patients took place in two stages: in the first phase, the patients were selected on the basis of age and diagnosis, using the tables compiled on the Breast Unit’s Excel file; in the second step, the digitized medical records were acquired, and the anamnestic data were collected. Based on such findings, an analysis of the diagnostic-therapeutic pathways of each patient was conducted, which enabled the authors to sift through the therapeutic protocols undertaken, thus determining whether reproductive counseling had been offered and eventually accepted/forgone. Such elements made it possible to shed light on the relationship between the diagnosis and the fertility preservation proposal and to investigate the possible discrepancy between counseling proposals and adherence to fertility preservation practices.

#### 2.2.3. Characteristics of the Sample

A total of 67 patients under the age of 40 diagnosed with malignant neoplasm were selected. The medical records examined completed the selection of patients. The age of 40 was chosen in light of the norms currently in place in the region, which limit access to oncofertility services to that age bracket. In addition, although this may be viewed as a limitation of the study, access to MAP techniques in the region through public hospitals are limited to patients ≤43. The 40-year ceiling was deemed appropriate by the authors due to the time that fertility treatment may take after the cancer diagnosis and the time needed for the news to sink in and for an informed decision to be made. The main characteristics of the patient sample as defined by inclusion/esclusion criteria are summarized in Table 1. Following a review of medical records, 51 patients were identified.

#### 2.2.4. Data Processing

The data collected through the review of medical records and the answers to the questionnaires were collected in a single spreadsheet using the Microsoft Excel program. The descriptive analysis, carried out ensuring the anonymity of the patients, was conducted using the same program for the calculation of the absolute and relative averages and frequencies.

## 3. Results

The data collected from the medical records are laid out below, divided according to the individual items searched.

**Figure 1:**
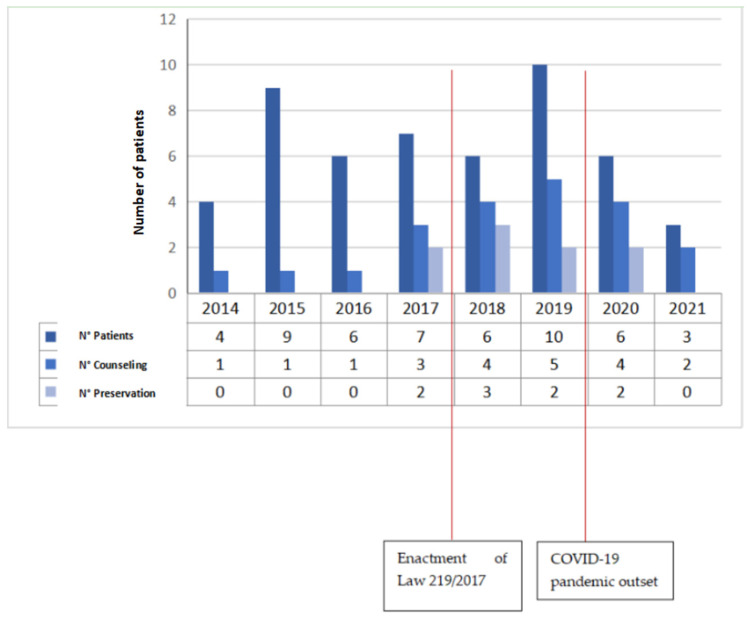
Number of patients, counseling interventions and fertility preservation procedures.

**Figure 2:**
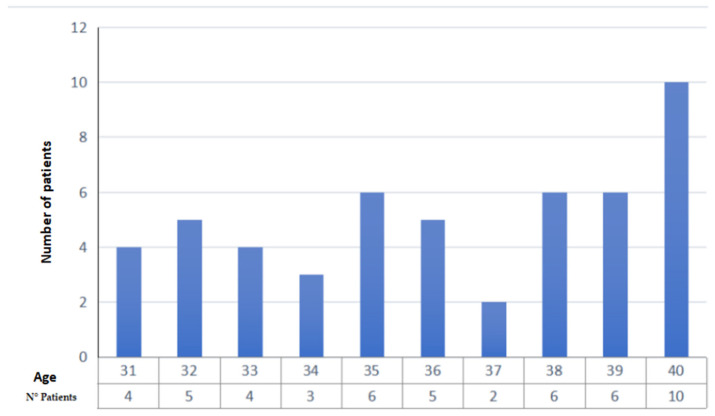
Age of patients at the time of diagnosis.

**Figure 3:**
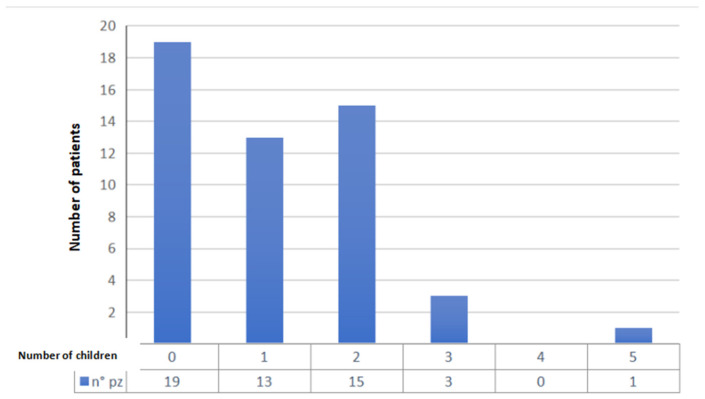
Patients subdivided according to the number of children at the time of diagnosis.

**Figure 4:**
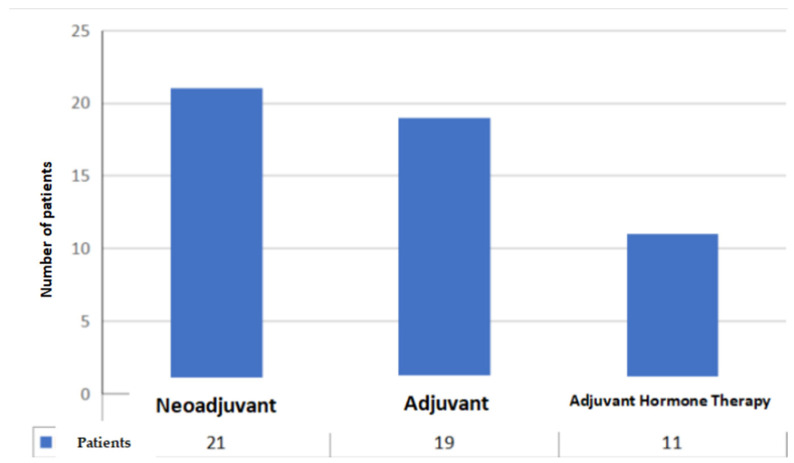
Type of therapeutic intervention undergone by patients.

**Figure 5:**
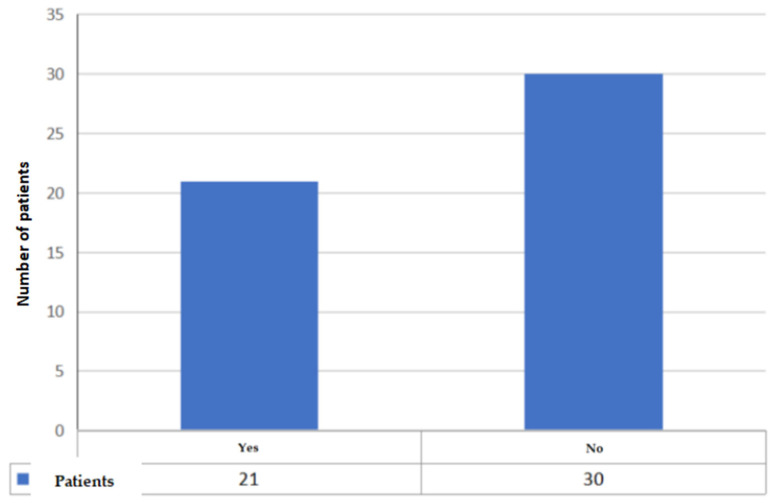
Number of patients who received counseling.

- Item 1: the first data collected are the year of diagnosis: in 2014, 4 patients were selected, in 2015, 9 patients were selected, in 2016, 6 patients were selected, 7 patients were selected in 2017, 6 in 2018, 10 in 2019, 6 in 2020, and 3 in 2021 (Figure 1). As is apparent, new and extraordinary obstacles to cancer care and oncofertility counseling have been caused by the COVID-19 pandemic [88]. Such challenges have affected access to fertility preservation procedures as well: elective procedures relying on ART have been discontinued or procrastinated, which are reflected in the study’s findings as well. The difficulties faced by patients as a result of the ongoing pandemic range from essential aspects such as gaining access to tertiary facilities performing both cancer treatments and fertility preservation interventions to the major concerns arising from the fear of possibly getting infected during fertility preservation procedures, which of course entail additional risks stemming from more hospital stays and surgical interventions. Hence, pandemic-related disruptions may even become a disincentivizing factor due to the sense of anxiety that some patients may experience out of fear of getting infected, which could even cause some patients to decide against fertility preservation despite their wish to start a family after cancer treatment. It is therefore of utmost importance for thorough oncofertility counseling to address the additional concerns and emotional distress experienced by many patients because of the ongoing pandemic while striving to outline and put in place additional targeted safety protocols in order to face the dangers and set the patients’ minds at ease [89].
- Item 2: the second parameter taken into consideration is the age of the patient, a primary inclusion criterion. Our study collected patients in the age group 31–40 years (mean age 36.15 years, standard deviation 3.0) (Figure 2);
- Item 3: the third data collected is the number of children at the time of diagnosis. A total of 19 patients had no children (37%), 13 had only 1 child (25%), 15 had 2 children (29%), 3 patients had 3 children (6%) and one patient had 5 children (2%) (Figure 3);
- Item 4: from the data collected regarding the therapy performed by the patients, it emerged that 21 patients (41%) underwent cycles of neoadjuvant chemotherapy, 19 patients (37%) underwent cycles of adjuvant chemotherapy and, for 11 patients (22%), adjuvant hormone therapy was prescribed (Figure 4);
- Items 5 to 8: relate to reproductive counseling and adherence to fertility preservation programs. Reproductive counseling was offered to 21 patients (41%); in the only cases reported (11), it was done through an interview (Figure 5).

**Figure 6:**
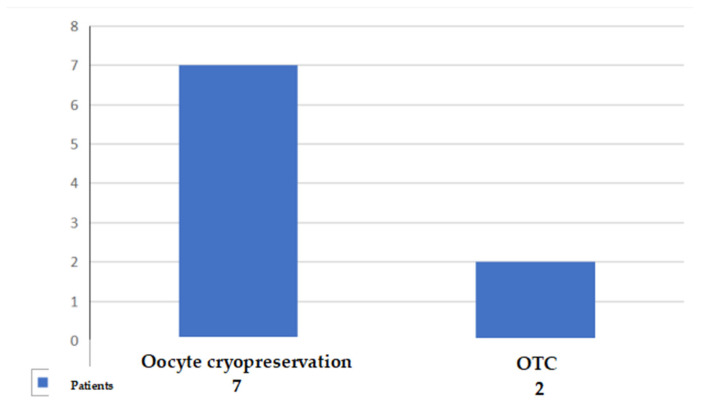
Patients broken down by technique of choice.

Out of the 21 patients who were offered the option to undergo fertility preservation interventions, 9 patients (43%) decided to accept it, while the remaining 12 patients (57%) did not. A total of 7 patients (78%) opted for oocyte cryopreservation, and 2 (22%) opted for ovarian tissue cryopreservation (OTC) (Figure 6).

## 4. Discussion

At present, thanks to the progress of increasingly sensitive diagnostic techniques and increasingly advanced and personalized therapeutic options, long-term survival in cancer patients in Italy has reached a rate of 63% at 5 years for women, most of which are breast cancer, considered to be the most frequent neoplasm in women [3]. Doctors’ attention is therefore shifting towards the quality of life of survivors and their future prospects.

The European Society of Medical Oncology (ESMO), in collaboration with ASCO in 2004, drafted a document [90] in which they state that an oncologist specialist should be educated on fertility preservation techniques. Hence, the guidelines seem to be in agreement on the need to start a discussion on the topic “oncofertility” early in order to jointly plan the antineoplastic treatment and the preservation of fertility.

Although it was conducted in a single facility, this study is in line with what emerged from the preliminary results of the already mentioned Italian PREFER study [61]. Moreover, the knowledge obtained from this study can be harnessed to set up research on larger samples. The collected data were subjected to a multivariate analysis to highlight any correlations between different parameters and graphically processed with the Microsoft Excel program.

The analysis of the data collected shows a poor knowledge of the topic of “oncofertility” by health personnel overall: of the 51 patients examined, only 21 (41%) received reproductive counseling. Nevertheless, by relating reproductive counseling to the period of interest, 2014–2021, a positive trend emerges both in absolute and relative terms, indicating a greater awareness of the issue by health personnel. In fact, since 2018, more than 60% of patients with indications for the preservation of fertility have received reproductive counseling, with a decrease in the number of cases in the last two years due to the pandemic.

By investigating the causes of the lack of counseling in the first years of the period of interest, the number of children at the time of diagnosis was related to the probability of having received counseling. It was found that, out of 21 women who were offered the opportunity to preserve fertility, 12 had no children (60%), 6 women had one child (28%), 2 women had 2 children (10%) and one woman had 3 children (5%). Analyzing the average age of the patients who received counseling, it is clear that young age may be a factor that prompts the specialist to offer counseling. In fact, the average age of all patients amounts to 36.1 years, which drops to 34.5 among those proposed to preserve fertility.

By analyzing the sample of 21 patients who received counseling, the authors have set out to investigate the causes that led them to accept or refuse the opportunity to preserve their fertility before antineoplastic therapy. In this case, the parameters to be taken into consideration are beyond the age of the patient and the number of children at the diagnosis; the insufficient information and scarce support in the decision-making process must also be taken into account. In fact, 12 patients (55%) felt the information provided by the healthcare staff has not been all-inclusive or sufficient to be able to make a conscious and informed decision, and 14 (67%) reported not feeling supported in the decision-making process; moreover, 11 chose to forgo fertility preservation due to concern for one’s health.

Although the overall figure is perfectly consistent with what emerges from the international literature, from the data relating to the year 2018, it can be deduced that considerable progress has been made in respecting national guidelines. However, the result of this study is an encouragement to keep moving in the right direction. Despite the indications issued by the oncofertility societies, there is no reproductive medicine specialist within the Breast Unit. Although the patients are referred to a center specialized in fertility preservation, an interview with a reproductive specialist as part of the multidisciplinary team that takes care of the cancer patient would guarantee better quality counseling, particularly for patients who express doubts about the usefulness and on the effectiveness of the treatment. Moreover, the patient would have the opportunity to discuss, over the course of the entire therapeutic process, with a specialist in the sector. This is the key to improve the quality of life of the patient during the course of her own history of disease. It is therefore easy to understand the fundamental role that the figure of a psychologist would play within the multi-specialist team, since both the neoplastic pathology and the risk of infertility are sources of major distress [91,92,93].

This study represents an initial phase of a university project on a multicentre scale. Alongside the review of the medical records, the research team drew up a questionnaire, specially designed for the group of selected patients, to be submitted by email to the aforementioned prior telephone contact. Alongside the questionnaire, a consent form was sent to participate in the study pursuant to the current legislation for the right to privacy [94].

The proposed questionnaire and form are available on request from the corrsponding author.

The questionnaire consisted of 14 items:

- Question 1 is, in turn, made up of 4 questions for the collection of general information on the patient at the time of diagnosis and at present;
- Question 2 asks for the diagnosis received;
- Questions 3 and 4 investigate the patient’s knowledge of the topic “oncofertility”;
- Questions 5 to 10 investigate the implementation of reproductive counseling, the methods of carrying out such counseling and the outcomes;
- Questions 11 and 12 are asked to find out whether the patient has had full-term pregnancies after recovery. If the patient answers affirmatively, they are asked if these pregnancies were natural or through MAP;
- Question 13 is addressed to patients who have not embarked on a fertility preservation process following reproductive counseling to investigate the causes;
- Question 14 is asked to patients who have not received counseling in order to know the patient’s interest in the possibility of preserving their fertility.

A recent publication by the regional Latium section of AIOM [95] reports the results of a survey carried out by interviewing various specialists (oncologists, radiotherapists, psychologists, gynecologists, oncologists) on the topic of “oncofertility” and fertility preservation techniques. From the responses received, it appears that only 25% of the interviewees are completely interested in the preservation of reproductive health and that more than a third of them have never offered reproductive counseling, while half have done so only sporadically. They justify their shortcomings with the impossibility of delaying the start of therapies, the lack of knowledge on the subject and the lack of experience in discussing the issue. Finally, doctors believe that the failure to comply with the indications of the guidelines is dictated by the lack of information, the absence of dedicated assistance, the embarrassment caused by dealing with an extremely private matter of the patient and the lack of a specialist psychologist available at the home center.

### 4.1. Medical-Legal Remarks

The fundamental role of reproductive counseling in the multidisciplinary management of the cancer patient is recognized by the major national scientific societies (AIOM-SIE-SIGO) [68] and international societies (ASCO) [90]. It also finds significant recognition in a legal-ethical context of rights and duties that any health professional must fulfill to the best of their abilities, and which, as a whole, constitute the medical act, whose lawfulness is codified in informed consent.

Although medical activity is acknowledged by the Italian Constitution, as health is one of the fundamental rights enshrined in that document, healthcare personnel are not authorized to carry out any clinical intervention without patient consent. Reproductive counseling thus comes to constitute an essential information phase to provide women with sufficient knowledge about the risks associated with therapy and fertility preservation strategies in order to acquire their informed consent. Hence, the lack of adequately thorough counseling invalidates the whole informed consent process.

In June 1992, the National Committee for Bioethics (NBC), in the document “Information and consent to the medical act” [96], highlighted that consent, acquired in writing, is a moral duty of the doctor for all those services which, for short and long term risks, duration, and presence of alternatives, require tangible proof that documents the patient’s will. The NBC specifies what the requisite distinctive traits of consent must be, to be properly verified and documented:

- Informed, hence based on exhaustive information which must be clear, appropriate to the educational level, age and cognitive functions of each patient, so that they are able to understand the diagnosis, the methods of intervention, the possible alternative therapies, the prognosis, the probabilities of success, the consequences and side effects of the treatment and the strategies to counter the latter;
- Granted in awareness, i.e., expressed by patients who, after having received thorough information, are enabled to understand and make informed decisions;
- Unequivocal, so as not to raise doubts or uncertainty as to the consent or refusal of the proposed treatment;
- Specific, i.e., strictly related to a specific treatment;
- Revocable at any time, even in proximity of the therapeutic act, as long as it is technically possible to refuse it. The withdrawal of consent must be recorded in the medical record in the same manner in which consent to the treatment was acquired;
- Free, i.e., not extorted by deception or duress, since only consent granted without undue external pressure can be considered valid;
- Personal, that is, it must come from patients in possession of their ability to act and to dispose of their bodies, or in any case from a guardian or legal representative holding power of attorney;
- Free of charge, since it cannot be granted in exchange for money or services of any kind.

In keeping with the standards laid out above, the 1992 document attributes fundamental value to the will of the patient, since personal freedom is enshrined in the Italian Constitution, which also upholds the right to self-determination on the basis of articles 2 and 13.

It is by those very foundational principles that Italian Law 219/2017 [97] (denominated “Rules on informed consent and advance processing provisions”) was enacted on 31 January 2018 in order to uphold “the right to life, health, dignity and self-determination of the person”. In addition, the legislation established that no health treatment can be initiated or continued without free and informed patient consent, except under extraordinary circumstances to be determined by law, in compliance with the earlier mentioned articles 2, 13 and 39 of the Italian Constitution [98] and articles 1, 2 and 3 of the Charter of Fundamental Rights of the European Union. It is therefore the ethical and legal duty of doctors to inform the patient as to the nature of possible therapeutic and prognostic options, risks and alternatives in order to protect the individual inalienable rights to health and self-determination. It should be pointed out that the term “risks” may encompass, in a broad sense, both health care costs and the threat of a deterioration in the quality of life of which the patient has to be informed.

At the same time, Italy’s Code of Medical Ethics, last updated in 2018, acknowledging the directives of the NBC and the provisions of the law, provides for the patient’s consent to undergo a diagnostic-therapeutic procedure. Article 33 of the code, following the provisions of law 219/2017, states that the doctor is charged with thoroughly informing the patient, or their legal guardians, on prevention, diagnostic procedures, prognosis, therapeutic options and any diagnostic-therapeutic alternatives. Moreover, all foreseeable risks and complications need to be discussed as well, in addition to behavioral indications that the patient will have to abide by over the course of the therapeutic process [99,100,101].

Although there is a growing trend in the interest of doctors on the topic of “oncofertility” and in line with medico-legal activity, the importance of the accurate implementation of reproductive counseling needs to be stressed, both for the prevention of litigation (since compliance with the guidelines has become a duty regulated in Italy by law 24/2017), and for the management of clinical risk, but also as an essential means to achieve the primary objective of the medical professional, namely, the care of the person as a whole.

### 4.2. Characteristics of the Study, Limitations and Future Prospects

Our study takes into consideration a sample of 51 patients aged between 31 and 40 from a single hospital center (the Breast Unit of Policlinico Umberto I), a small sample which could be scarcely representative of the entire patient population and of the population of women diagnosed with breast cancer overall. In addition, the 40 year cut-off for patients was chosen in light of the current regulations governing oncofertility and MAP access in the Italian region where the study was conducted, although that may be seen as a limitation as well. However, the findings herein are in line with some outcomes of one of the largest reviews in this area, which takes into consideration 147 studies conducted in 20 different countries [90].

In particular, relevant shared conclusions highlighted the following points:

- The need for more information to patients, both oral and, above all, written, through documents and printed resources;
- The need for greater psychological support and assistance to the patient in the decision to preserve fertility;
- The need for greater training of health personnel in this area;
- A positive trend has emerged in recent years with respect to oncofertility information provision to patients.

On the last point, this positive trend is confirmed in the very history of fertility preservation: it is worth highlighting how the cryopreservation of oocytes is no longer viewed as an experimental technique, while the cryopreservation of ovarian tissue is still considered as such.

In addition, the joint recommendations of AIOM, SIE and SIGO on the establishment of oncofertility centers date back to 2016. Currently, 28 public centers for cryopreservation are fully operational in Italy, mostly in the North. It is therefore quite clear that the fundamental mission of the health personnel of the Breast Unit of the Policlinico Umberto I is in keeping with the evolution of the oncofertility theme at the national level.

The primary objective of our study is the evaluation of the clinical activity carried out by the multidisciplinary team providing care for breast cancer patients, with a close focus on how and to what extent health care personnel addresses the issues of reproductive health and fertility preservation. For the purpose of helping medical staff provide better care, it is useful to investigate the answers of patients in psychosocial terms rather than strictly clinical ones. To that end, a questionnaire was put together and submitted to the patients selected for this study in order to obtain a well-rounded knowledge of the subject from both the doctors’ and the patients’ perspectives.

## 5. Conclusions

This study was meant to highlight the trend in modern society to postpone motherhood and the progress in reproductive medicine, as well as the importance of adequate reproductive counseling, which aimed at reconciling the cancer diagnosis with the preservation of the woman’s reproductive health. The creation of a program for the protection of reproductive health is supported by the activity of forensic medicine, whose primary objective is to guarantee adherence to national and international guidelines and to the recommendations for the preservation of fertility in cancer patients. Forensic medicine is therefore essentially preventive, favoring the reduction of litigation and improving clinical risk management. There are three main issues of considerable medico-legal relevance: the non-compliance with or mismanagement of reproductive counseling, the acquisition of consent that respects all the attributes that characterize it and the lawfulness of the fertility preservation and MAP techniques, regulated in Italy by law 40/2004. Although it is quite understandable for both doctors and patients to focus on the path to recovery, it is necessary to inform the patient, preferably at the time of diagnosis, as to the existence of various options for fertility preservation in order to foster freedom of choice. This clinical practice is, in fact, both a legal and ethical obligation of the healthcare professional based on core provisions of Italian law 219/2017 and the National Code of Medical Ethics.

Despite the interest and the sensitivity exhibited by many health care institutions such as the Breast Unit of Rome’s Policlinico Umberto I, there is still a long way to go in the development of a standardized and evidence-based diagnostic-therapeutic protocol for reproductive counseling, in accordance with the 2016 AIOM-SIE-SIGO recommendations, among others.

In conclusion, the authors feel that two fundamental recommendations from this study are especially noteworthy:

1. Create a multi-specialist team within each operating unit for the diagnosis and treatment of neoplastic diseases, which should include oncologists, surgeons, endocrinologists, gynecologists, psychologists and reproductive medicine specialists. The fundamental purpose and priority of the health care team is to fully cover and address all the various complexities and distinctive traits inherent in the oncofertility blueprint and to effectively address women’s psychosocial distress. The team must be able to provide reproductive counseling at the time of diagnosis or soon after the diagnostic-therapeutic process, since fertility, far from being a mere biological parameter, often constitutes a cornerstone of femininity for society and patients themselves;
2. Outline and implement a single and standardized reproductive counseling protocol, laid out and agreed on by highly qualified specialists, documented in medical records with informed consent value, based on what has been determined by legal regulations and medical ethics provisions.

## Figures and Tables

**Table 1:** Patient sample characteristics.

Patient Sample Characteristics	
Inclusion Criteria	Exclusion Criteria
Age ≤ 40 (ultimate average age was 36.15; standard deviation: 3.0)	Previous diagnosis of malignancy, breast or otherwise
Diagnosed between 2014 and 2021	Previous diagnosis of metastatic disease [86,87]
History of neoadjuvant or adjuvant chemotherapy or hormone therapy	

## Data Availability

The data presented in this study are available on request from the corresponding author.
